# Consumption and Dependence on Benzodiazepines in Elderly Outpatients in Psychiatry

**DOI:** 10.1192/j.eurpsy.2026.11593

**Published:** 2026-07-31

**Authors:** N. Smaoui, M. Barkallah, S. Omri, I. Gassara, R. Feki, M. Maalej, N. Charfi, L. Zouari

**Affiliations:** Psychiatry Department “C”, Hedi Chaker Hospital, Sfax, Tunisia

## Abstract

**Introduction:**

Chronic use of benzodiazepines (BZD) among the elderly is a major public health concern due to the risk of dependence, cognitive decline, and psychiatric comorbidities.

**Objectives:**

We aimed to describe the consumption patterns and dependence profile of elderly psychiatric outpatients using BZD.

**Methods:**

A cross-sectional study was conducted among patients aged 65 years and older attending outpatient psychiatric consultations. Data collected included benzodiazepine use, attempts at withdrawal, concomitant medications, and substance use. Psychometric assessments were performed using the Benzodiazepine Attachment Scale (ECAB), the Hospital Anxiety and Depression Scale (HADS-A and HADS-D), and the Mini-Mental State Examination (MMSE) to assess cognitive function.

**Results:**

Seventy-three patients were included (male/female ratio = 1.08). Psychoactive substance use was reported by 81% of patients, and polysubstance use by 41%. BZD consumption was found in 69% of cases (n = 51), with a mean duration of use of 23.3 years (range: 2–40 years). The most frequently used molecule was lorazepam, reported in 61% of cases. During the six months preceding the study, BZD use was daily in 92% of cases. Among the 51 BZD users, 31 patients (61%) had attempted withdrawal; among them, 27 (87%) reported failure. The main reasons for withdrawal failure were withdrawal syndrome (48%) and reappearance of initial symptoms (44%).

Among the 51 patients who used BZD, 41 (80%) met the criteria for dependence. A significant association was observed between BZD dependence and living in urban or semi-urban areas (p = 0.04). Clinically, dependence was significantly linked to the presence of personal somatic history (p = 0.021), depressive and bipolar disorders (p = 0.024), younger age at onset of psychiatric illness (p = 0.05), and longer illness duration (p = 0.035).

Regarding substance use, BZD dependence correlated with caffeine use (p = 0.021), stimulant use (p = 0.024), and polysubstance use (p = 0.038). Strong associations were also found with the duration (p < 0.001) and frequency (p = 0.021) of BZD use, as well as with failed withdrawal attempts (p < 0.001). On psychometric assessment, dependent patients showed significantly higher HADS-D (p = 0.001) and HADS-A (p = 0.005) scores, indicating more severe depressive and anxious symptomatology. Regarding cognitive performance, a negative correlation was observed between age (in years) and MMSE score among dependent patients (p = 0.001), while this association was less pronounced in non-dependent patients (p = 0.016).

**Conclusions:**

BZD dependence in the elderly is associated with marked anxiety-depressive symptoms and cognitive decline. These findings highlight the importance of systematic assessment of dependence, mood, and cognitive function in elderly BZD users, to implement integrated, gradual, and tailored management strategies for this vulnerable population.

**Disclosure of Interest:**

None Declared

